# Immunogenicity and safety of inactivated SARS-CoV-2 vaccine (CoronaVac) using two-dose primary protocol in children and adolescents (Immunita-002, Brazil): A phase IV six-month follow up

**DOI:** 10.21203/rs.3.rs-3931021/v1

**Published:** 2024-02-29

**Authors:** Camila Amormino Corsini, Priscila Fernanda da Silva Martins, Priscilla Soares Filgueiras, Adelina Júnia Lourenço, Ana Esther de Souza Lima, Sarah Vieira Contin Gomes, Wander de Jesus Jeremias, Pedro Augusto Alves, Gabriel da Rocha Fernandes, Luciana Lisboa Mota e Castro, Andrea Teixeira de Carvalho, Ana Carolina Campi Azevedo, Caroline De Almeida Leitao Curimbaba, Daniela Aparecida Lorencini, Eolo Morandi Junior, Victor Mattos da Silva, Maria Célia Cervi, Marcos de Carvalho Borges, Maurício Lacerda Nogueira, Guilherme Rodrigues Fernandes Campos, Paulo Roberto Lopes Correa, Taciana Malheiros Lima Carvalho, Jordana Grazziela Alves Coelho dos Reis, Erik Vinícius de Sousa Reis, Leda dos Reis Castilho, Poliana Remundini de Lima, João Paulo Resende do Nascimento, Jaquelline Germano de Oliveira, Olindo Assis Martins Filho, Rafaella Fortini Queiroz e Grenfell, Immunita team

**Affiliations:** 1 Oswaldo Cruz Foundation (FIOCRUZ). 1715 Augusto de Lima Avenue, Belo Horizonte, Minas Gerais, Brazil. 30190-002; 2 Department of Pharmacy, Federal University of Ouro Preto (UFOP). 27, Nove, Bauxita, Ouro Preto, Brazil. 35400-000; 3 Instituto Butantan, São Paulo, São Paulo, Brazil. 05503-900; 4 Faculty of Medicine, University of São Paulo (USP). 455 Doutor Arnaldo Avenue, São Paulo, São Paulo, Brazil. 01246-903; 5 Serrana Clinical Research Center. 438, 13 de Maio, Centro, Serrana, São Paulo, Brazil. 14150-000; 6 Faculty of Medicine of São José do Rio Preto (FAMERP). 5416 Brigadeiro Faria Lima Avenue, São José do Rio Preto, São Paulo, Brazil. 15090-000; 7 Hospital de Base, 5544 Brigadeiro Faria Lima Avenue, São José do Rio Preto, São Paulo State, Brazil. 15090-000; 8 Department of Pathology, University of Texas Medical Branch, 301 University Blvd, Galveston, TX, USA. 77555; 9 Belo Horizonte Municipal Health Department (SMS), 2336 Afonso Pena Avenue, Belo Horizonte, Brazil. 30130-012; 10 Federal University of Minas Gerais (UFMG). 6627 Presidente Antônio Carlos Avenue, Belo Horizonte, Minas Gerais, Brazil. 31270-901; 11 Cell Culture Engineering Laboratory (COPPE), Federal University of Rio de Janeiro (UFRJ). 550 Pedro Calmon Avenue, Rio de Janeiro, Rio de Janeiro, Brazil. 21941-598; 12 Department of Infectious Diseases, College of Veterinary Medicine, University of Georgia (UGA). 501 DW Brooks Drive, Athens, Georgia, USA. 30602-7387

**Keywords:** Vaccine, CoronaVac, SARS-CoV-2, COVID-19, neutralizing antibodies, antibodies kinetics, cellular markers

## Abstract

**Introduction:**

Vaccines are essential for the prevention and control of several diseases, indeed, monitoring the immune response generated by vaccines is crucial. The immune response generated by vaccination against SARS-CoV-2 in children and adolescents is not well defined regarding to the intensity and medium to long-term duration of a protective immune response, which may point out the need of booster doses and might support the decisions in public health.

**Objective:**

The study aims to evaluate the immunogenicity and safety of inactivated SARS-CoV-2 vaccine (CoronaVac) in a two-dose primary protocol in children and adolescent aging from 3 to 17 years old in Brazil.

**Methods:**

Participants were invited to participate in the research at two public healthcare centers located in Serrana (São Paulo) and Belo Horizonte (Minas Gerais), Brazil. Participants underwent medical interviews to gather their medical history, including COVID-19 history and medical records. Physical exams were conducted, including weight, blood pressure, temperature, and pulse rate measurements. Blood samples were obtained from the participants before vaccination, 1 month after the first dose, and 1, 3, and 6 months after the second dose and were followed by a virtual platform for monitoring post-vaccination reactions and symptoms of COVID-19. SARS-CoV-2 genome from Swab samples of COVID-19 positive individuals were sequenced by NGS. Total antibodies were measured by ELISA and neutralizing antibodies to B.1 lineage and Omicron variant (BA.1) quantified by PRNT and VNT. The cellular immune response was evaluated by flow cytometry by the quantification of systemic soluble immune mediators.

**Results:**

The follow-up of 640 participants showed that the CoronaVac vaccine (Sinovac/Butantan Institute) was able to significantly induce the production of total IgG antibodies to SARS-CoV-2 and the production of neutralizing antibodies to B.1 lineage and Omicron variant. In addition, a robust cellular immune response was observed with wide release of pro-inflammatory and regulatory mediators in the early post-immunization moments. Adverse events recorded so far have been mild and transient except for seven serious adverse events reported on VigiMed.

**Conclusions:**

The results indicate a robust and sustained immune response induced by the CoronaVac vaccine in children and adolescents up to six months, providing evidences to support the safety and immunogenicity of this effective immunizer.

## 1. INTRODUCTION

The development of safe and effective vaccines to SARS-CoV-2 is crucial for managing the COVID-19 pandemic and will continue to be the primary tool for limiting the virus’s spread (GRENFELL et al., 2022). However, the immune response generated by COVID-19 vaccines are still under investigation, and further studies are necessary to gain a better understanding of the protection to SARS-CoV-2 upon vaccination, especially in children and adolescents. In this age group, the immune response triggered by vaccination is not clearly defined in terms of its intensity and duration of protection in the medium and long term, as well as its ability to neutralize distinct variants of concern (VOCs). This information may indicate the need for booster shots and require healthcare managers to make informed decisions (WALTER et al., 2022).

The aim of the present study was to comprehensively assess both humoral and cellular responses and evaluating the effectiveness of the two-dose primary protocol of CoronaVac vaccination in children and adolescents in Brazil. This investigation encompassed the identification of positive COVID-19 cases post-vaccination, infection severity, clinical data, and outcomes in children and adolescents aged 3 to 17 who received the two-dose primary protocol of CoronaVac vaccine (Sinovac/Butantan Institute) against COVID-19 over a six-month period. The data generated throughout this phase 4 monitoring study in children and adolescents will contribute significantly to enhance our understanding of the protective response, effectiveness, and safety of COVID-19 vaccines in this specific age group.

## 2. METHODS

### 2.1 Ethics statement and participants

This study received the approval from the Research Ethics Committee involving Human Subjects at the Oswaldo Cruz Foundation, the Ethics Committee of Hospital das Clínicas da Faculdade de Medicina de Ribeirão Preto – University of São Paulo, and the National Council of Ethics in Research (CAAE 55183322.6.0000.5091). Inclusion criteria comprised children and adolescents aged 3 to 17 years, unvaccinated for COVID-19, who, in agreement with their parents or guardians, willingly participated in the study and signed the informed consent and assent forms (ICF/IAF). Informed consent was obtained from the parent(s) and/or legal guardian(s) of all minor participants involved in this study. The consent process included a detailed explanation of the study purpose, procedures, potential risks, and benefits. Participants were informed that their participation was voluntary, and they had the right to withdraw at any time without penalty. Parent(s) and/or legal guardian(s) were provided with contact information for any questions or concerns regarding the study. As for the deferral criteria, participants with suspected SARS-CoV-2 infection waited up to 72 hours for confirmation of the diagnosis. When confirmed, vaccination was postponed for a minimum of four weeks. All methods described in this study were performed in accordance with the relevant guidelines and regulations.

### 2.2 Participant recruitment, sample collection, and follow-up

Participants were invited to participate in the research at two public healthcare centers located in Serrana, São Paulo, and in Belo Horizonte, Minas Gerais, Brazil. Following the acquisition of informed consent and the application of inclusion and deferral criteria, each participant, along with their parents or guardians, underwent an interview with the medical team to obtain a comprehensive medical history, any previous history of COVID-19 infections, and information regarding concurrent medications, whether related or unrelated to their medical history.

After, physical examinations were conducted, encompassing measurements of weight and vital signs such as systolic and diastolic blood pressure, axillary temperature, and pulse rate. After the completion of these examinations, the participants underwent peripheral blood sampling and were then guided through the vaccination procedure within the healthcare facility.

A total of 640 participants who met the inclusion criteria were followed for a duration of six months after completing the two-dose primary protocol of CoronaVac vaccine (Sinovac, Butantan Institute), administered with a 28-day interval between doses.

Peripheral blood samples were collected at multiple time points: prior to vaccination, on the day of the second dose administration, and at one month, three months, and six months post-second dose, with reference to the date of administering the second dose of the CoronaVac vaccine (Sinovac, Butantan Institute), as well as carrying out the physical examinations described previously. One sample of 10 ml of whole blood were obtained via venous puncture from each participant according to biosafety standards and subsequently centrifuged at 3,000 g/5 min to obtain serum for immunogenicity analyses.

**Figure 1.**
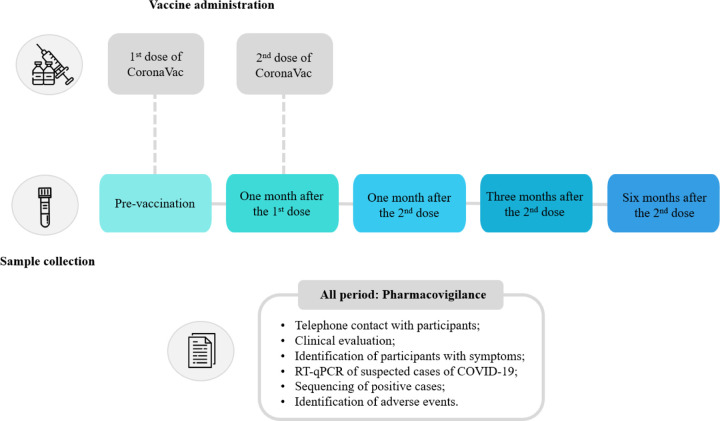
Schematic representation of the Immunita-002 study design.

All participants were daily monitored through a virtual platform for a duration of seven days to report any post-vaccination adverse events, and continuously for the reporting of any suspected COVID-19 symptoms. Those displaying symptoms suggestive of the disease underwent a medical evaluation, which included nasopharyngeal swab collection to confirm the diagnosis via RT-qPCR. Positive samples from these individuals underwent next-generation sequencing (NGS) for further analysis. Data regarding participants’ hospitalizations or adverse events were obtained from medical reports and/or the participants’ medical records. The study design is illustrated in the schematic representation of Figure 1.

### 2.3 Assessment of anti-S and anti-N IgG antibodies and viral neutralization assays to SARS-CoV-2

All serum samples obtained from individuals participating in the study were subjected to testing for specific total IgG antibodies to Spike (S) and Nucleocapsid (N) proteins of SARS-CoV-2 at all study sample collection times. These proteins, used as antigens, were generated in stable recombinant HEK293 cells, as outlined in the work of ALVIM et al. (2020). The detection of antibodies was carried out using standardized ELISA assays, in accordance with the methodology established by GRENFELL et al. (2022), which had been validated by the National Institute of Health Quality Control of Oswaldo Cruz Foundation (INCQS/Fiocruz).

For the assessment of SARS-CoV-2 neutralizing antibodies, a subset of participants who had not previously been diagnosed with COVID-19 was evaluated at multiple time points: before vaccination, and at one month, three months, and six months post-second dose of the CoronaVac vaccine. Two distinct assays were employed for this purpose: the plaque reduction neutralization test (PRNT) assessed neutralizing antibodies to the SARS-CoV-2 (B.1), while the viral microneutralization assay (VNT50) evaluated neutralizing antibodies against the Omicron variant (BA.1). These assessments were conducted following the protocols outlined by GRENFELL et al. (2022) and CAMPOS et al. (2022).

### 2.4 Quantification of immune soluble mediators

The Luminex Bio-Plex Pro^™^ human cytokines platform was used to quantify systemic soluble biomarkers, allowing the investigation of 27 analytes, including chemokines (CXCL8, CCL11, CCL3, CCL4, CCL2, CCL5 and CXCL10), pro-inflammatory cytokines (IL-1β, IL-6, TNF-α, IL-12, IFN-γ, IL-15 and IL-17), regulatory cytokines (IL-1Ra, IL-4, IL-5, IL-9, IL-10 and IL-13), and growth factors (FGF-basic, PDGF, VEGF, G-CSF, GM-CSF, IL-7 and IL-2). A total of 173 samples from children and adolescents with no previous diagnosis of COVID-19 were assessed along the kinetics timeline, comprising: before vaccination, one month, three months, and six months after the second dose of the CoronaVac vaccine. Measurements were conducted using a Bio-Plex 200 instrument (Bio-Rad).

### 2.5 Identification of adverse events and serious adverse events following vaccine administration

All participants underwent a seven-day monitoring following the administration of each dose of the COVID-19 vaccine, which was facilitated through telephone communication, with the aim of identifying any adverse events (AEs) and serious adverse events (SAEs).

In this study, AEs encompassed any unfavorable medical incidents reported by vaccinated participants, which may not necessarily have had a direct causal connection with vaccine administration. In contrast, SAEs were defined as any adverse event leading to one of the following outcomes: death, risk of death at the time of the event, hospitalization or extension of existing hospitalization, significant or persistent disability that significantly disrupted an individual’s ability to carry out routine functions, or clinically significant events arising from the use of necessary medications during a medical intervention aimed at preventing death, significant disability, or hospitalization of the participant.

During in-person follow-up visits and within the initial seven days following each vaccine dose, participants were queried about the occurrence of specific signs and symptoms (solicited AEs), as well as the spontaneous reporting of additional signs and symptoms (unsolicited AEs). The intensity of solicited AEs was categorized using a numerical scale ranging from 1 to 4, as detailed in Supplementary table 1 (local events) and Supplementary table 2 (systemic events), developed in accordance with the Toxicity Grading Scale for Healthy Adult and Adolescent Volunteers Enrolled in Preventive Vaccine Clinical Trials by the United States Food and Drug Administration (FDA, USA) and the Common Terminology Criteria for Adverse Events - Version 5.0 by the United States National Cancer Institute (NCI/NIH, USA). Similarly, unsolicited AEs were graded based on this numerical scale, as per Supplementary table 3, in line with the same guidelines. The highest reported intensity for an AE, until its resolution or outcome, was employed for study analyses. Furthermore, AEs were classified based on their causal relationship with the CoronaVac vaccine, following the adapted classification from the Uppsala Monitoring Centre of the World Health Organization (UMC, WHO), as outlined in Supplementary table 4. All local reactions following vaccination were considered AEs with a definitive causal connection to the vaccine. All solicited and unsolicited AEs identified during the initial two weeks after each vaccine dose’s administration were documented, irrespective of their causal association with the vaccine. These identified events were meticulously recorded in a spreadsheet utilizing the RedCap software, with immediate notification of SAEs to the VigiMed platform of the Brazilian National Health Surveillance Agency (Anvisa, Brazil).

### 2.6 Identification of COVID-19 positive cases by RT-qPCR and subsequent SARS-CoV-2 sequencing by NGS

In case of suspected COVID-19 during the study period, purified RNA from nasopharyngeal swab samples of individuals was used for RT-qPCR using the Charité/Berlin and Centers for Disease Control and Prevention (CDC, USA) protocols (CORMAN, V. et al., 2019). SARS-CoV-2 positive samples with a cycle threshold (Ct) value < 34 were subjected to NGS sequencing, following the protocol described by GRENFELL et al. (2022) and DEZORDI et al. (2022). The obtained sequences were promptly deposited in the Global Initiative on Sharing All Influenza Databank (GISAID).

### 2.7 Statistical analysis and data mining

Data analyses were conducted employing GraphPad Prism^®^ software. The results obtained from antibody titer quantification were subjected to statistical analysis utilizing the Kruskal-Wallis test and Mann-Whitney normality tests, with a significance level set at p < 0.05. Signatures of soluble mediators were derived by transforming serum levels, initially expressed as continuous variables (pg/mL). For the clinical monitoring records, the platforms Cytoscape and RedCap were employed.

## 3. RESULTS

### 3.1 Demographic data of the patients

A total of 640 participants ranged in age from 3 to 17 years old (median 9 years) were consented and enrolled in the study. Samples were collected from March 2022 to July 2023. A total of 325 (50.75%) of the participants were male and 315 (49.22%) were female. The most common pre-existing comorbidities were rhinitis 73 (11.41%), asthma 42 (6.56%), obesity 18 (2.81%), sinusitis 9 (1.41%), bronchiolitis 8 (1.25%), and bronchitis 8 (1.25%), as described in Table 1.

### 3.2 Immunogenicity response: Kinetics of total anti-S and anti-N IgG antibodies, neutralizing antibodies to live SArS-COV-2, and immune soluble mediators

**Figure 2.**
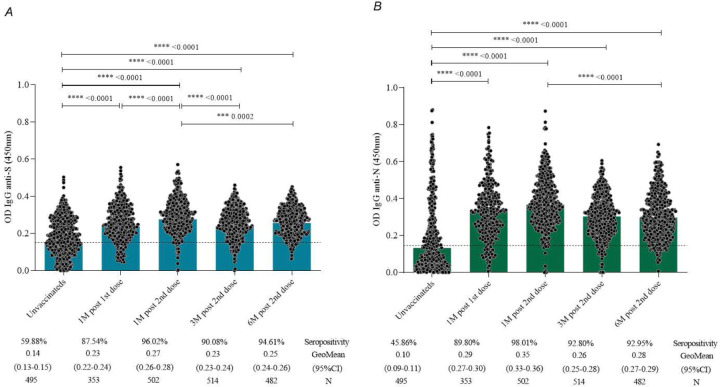
Kinetics of anti-S IgG (A) and anti-N IgG (B) levels of SARS-CoV-2 at pre-vaccination, 1 month after the first dose, and 1 month, 3 months, and 6 months after receiving the second dose of the CoronaVac vaccine (Sinovac/Butantan Institute). The detection limit of 0.1508 in (A) and 0.1460 in (B) is represented by dashed lines. The black dots represent individual data points of optical density (450nm) for each vaccinated participant. The percentage values indicate the seropositivity rate. The geometric mean of IgG anti-S and anti-N antibody titers is represented by blue and green bars, respectively. Statistical differences defined by Kruskal-Wallis and Mann-Whitney methods are represented for comparisons over time.

When evaluating the kinetics of total IgG antibodies against S and N proteins of SARS-CoV-2 (Figure 2), a significant difference in the titers of anti-S (p < 0.0001) and anti-N IgG antibodies (p < 0.0001) was observed one month after completing the primary immunization, and this difference persisted throughout the analyzed period of up to six months for both specific antibodies assessed. Seropositivity rates were 96.02%, 90.08%, and 94.61%, respectively, at 1, 3, and 6 months, when considering antibodies against the S protein of the virus. A similar profile was identified for the nucleocapsid protein, where seropositivity rates were 98.01%, 92.80%, and 92.95%, respectively, at 1, 3, and 6 months after receiving both doses of the CoronaVac vaccine.

**Figure 3.**
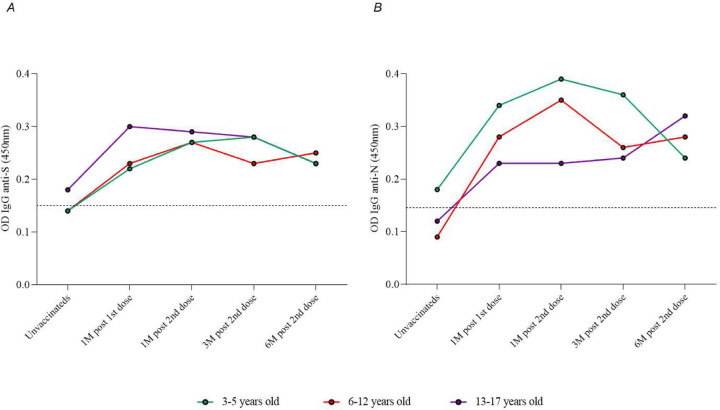
Kinetics of total IgG anti-S (A) and anti-N (B) antibody levels to SARS-CoV-2 at pre-vaccination, 1 month after the first dose, and 1 month, 3 months, and 6 months after receiving the second dose of the CoronaVac vaccine (Sinovac/Butantan Institute) separately for age groups (3–5, 6–12, and 13–17 years old). The detection limit of 0.1508 in (A) and 0.1460 in (B) is represented by dashed lines. The colored dots represent the geometric mean of optical density (450nm) for each vaccinated age group.

In the age group (3–5, 6–11, and 12–17 years old) analysis, the kinetics of total IgG antibodies to S and N proteins had the same response profile, with a statistically significant increase in total antibodies one month after the application of the second dose of the CoronaVac vaccine compared to pre-vaccination levels (Figure 3).

**Figure 4.**
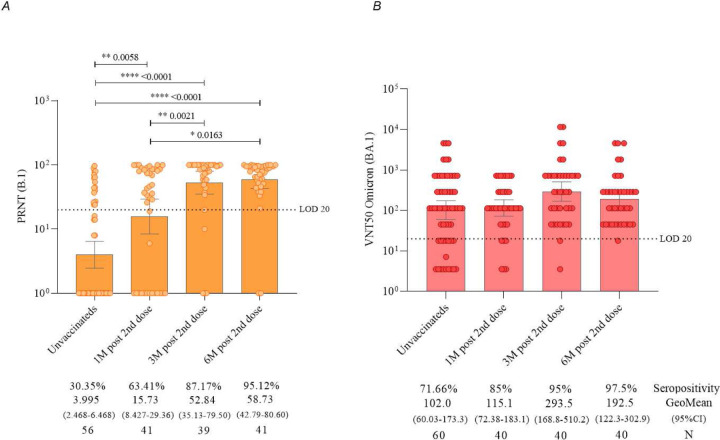
(A) Neutralizing antibodies detected by PRNT to B.1 lineage of SARS-CoV-2 in children and adolescents vaccinated with CoronaVac (Sinovac/Butantan Institute). The cutoff for seropositivity definition of 20 is represented by dashed lines. The geometric mean antibody titer is represented by orange bars. The colored points represent the individual result of each participant at different follow-up times in the study. Statistical differences defined by Mann-Whitney are presented for comparisons over time. (B) Neutralizing antibodies detected by VNT50 to Omicron variant (BA.1) of SARS-CoV-2 in children and adolescents vaccinated with CoronaVac (Sinovac/Butantan Institute). The cutoff for seropositivity definition of 20 is represented by dashed lines. The geometric mean antibody titer is represented by red bars. The colored points represent the individual result of each participant at different follow-up times in the study. Statistical differences defined by Mann-Whitney are presented for comparisons over time.

The levels of neutralizing antibodies determined by the PRNT assay using the B.1 lineage of SARS-CoV-2 (Figure 4A) demonstrated a significant increase in seropositivity rate was observed from 30.35% to 63.41% at 1 month, 87.17% at 3 months, and 95.24% at 6 months (p < 0.0001) after the administration of primary protocol. The mean assessment showed an increase of 108.93% at 1 month, 187.23% at 3 months, and 213.87% at 6 months after vaccination, compared to the initial time point of this study.

Regarding the titers of neutralizing antibodies determined by the VNT50 methodology with Omicron variant (BA.1) (Figure 4B), a seropositivity rate of 71.66% increased to 85% at 1 month, with a significant increase to 95% at 3 months (p = 0.0207), and 97.5% at 6 months after the application of the second dose of the CoronaVac vaccine. The mean assessment showed an increase of 18.61% at 1 month, 32.55% at 3 months, and 36.02% at 6 months after vaccination, compared to the first follow-up time.

**Figure 5.**
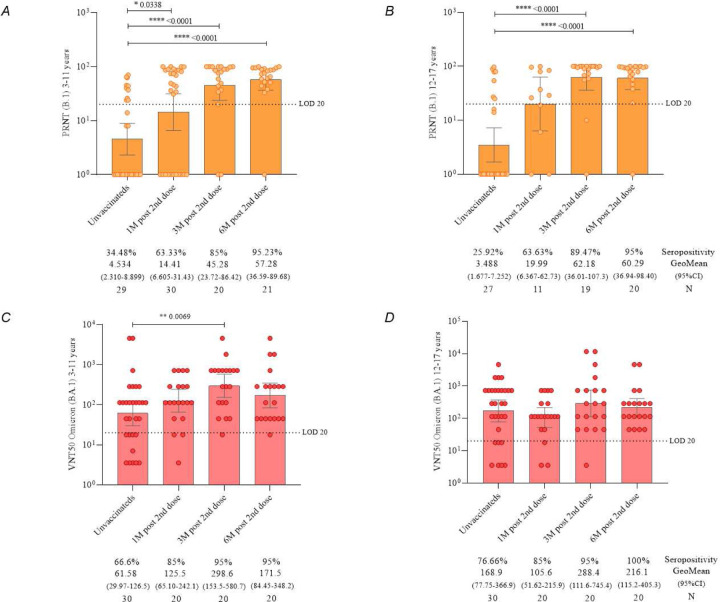
(A) Neutralizing antibodies detected by PRNT to B.1 lineage of SARS-CoV-2 in children aged 3 to 11 years vaccinated with CoronaVac (Sinovac/Butantan Institute). The cutoff for seropositivity definition of 20 is represented by dashed lines. The geometric mean antibody titer is represented by orange bars. The colored points represent the individual result of each participant at different follow-up times in the study. Statistical differences defined by Mann-Whitney are presented for comparisons over time. (B) Neutralizing antibodies detected by PRNT to B.1 lineage of SARS-CoV-2 in adolescents aged 12 to 17 years vaccinated with CoronaVac (Sinovac/Butantan Institute).

When analyzing neutralizing antibodies by age subgroups, both children (3–11 years) (Figure 5A) and adolescents (12–17 years) (Figure 5B) showed a significant increase in the detection of neutralizing antibodies to B.1 lineage of SARS-CoV-2 3 and 6 months after the second dose of the CoronaVac vaccine, compared to pre-vaccination levels. However, neutralizing antibodies to Omicron variant (BA.1) increased significantly 3 after the second dose of the CoronaVac vaccine (Figure 5, C and D), compared to pre-vaccination levels, only in children (p = 0.0069).

The cutoff for seropositivity definition of 20 is represented by dashed lines. The geometric mean antibody titer is represented by orange bars. The colored points represent the individual result of each participant at different follow-up times in the study. Statistical differences defined by Mann-Whitney are presented for comparisons over time. (C) Neutralizing antibodies detected by VNT50 to Omicron variant (BA.1) in children aging from 3 to 11 years vaccinated with CoronaVac (Sinovac/Butantan Institute). The cutoff for seropositivity definition of 20 is represented by dashed lines. The geometric mean antibody titer is represented by red bars. The colored points represent the individual result of each participant at different follow-up times in the study. Statistical differences defined by Mann-Whitney are presented for comparisons over time. (D) Neutralizing antibodies detected by VNT50 to Omicron variant (BA.1) in adolescents aging from 12 to 17 years vaccinated with CoronaVac (Sinovac/Butantan Institute). The cutoff for seropositivity definition of 20 is represented by dashed lines. The geometric mean antibody titer is represented by red bars. The colored points represent the individual result of each participant at different follow-up times in the study. Statistical differences defined by Mann-Whitney are presented for comparisons over time.

Regarding the evaluation of neutralizing antibodies against the Omicron variant (BA.1) in these same age groups (Figure 5, C and D), it was observed that only children exhibited a statistically significant difference in the pre-vaccination period compared to the three months post the administration of the second dose of CoronaVac (p = 0.0069).

**Figure 6.**
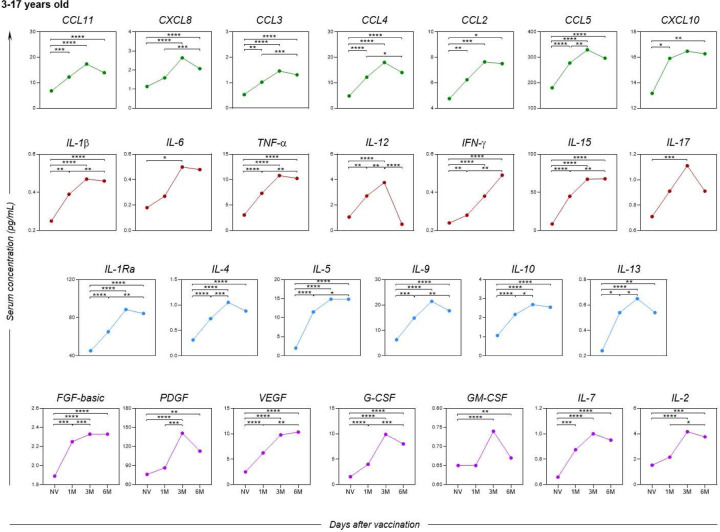
Kinetics of serum soluble mediators at one month (1M), three months (3M), and six months (6M) after receiving two doses of the CoronaVac vaccine in children and adolescents (3–17 years old) as compared to the pre-vaccination period (Not Vaccinated - NV). The biomarkers were individually presented for chemokines (CXCL8, CCL11, CCL3, CCL4, CCL2, CCL5 and CXCL10); pro-inflammatory cytokines (IL-1β, IL-6, TNF-α, IL-12, IFN-γ, IL-15 and IL-17); regulatory cytokines (IL-1Ra, IL-4, IL-5, IL-9, IL-10 and IL-13) and growth factors (FGF-basic, PDGF, VEGF, G-CSF, GM-CSF, IL-7 and IL-2). Statistical differences by Mann-Whitney and ANOVA with significance levels of p < 0.05 are denoted by (*) between groups.

Serum soluble mediators from participants who had no previous diagnosis of COVID-19 at pre-vaccination or during the 6-months follow up revealed an increase in the levels of chemokines (CCL11, CCL3, CCL4, CCL2, CCL5 and CCL10), pro-inflammatory cytokines (IL-1β, TNF-α, IL-12, IFN-γ and IL-15), regulatory cytokines (IL-1Ra, IL-4, IL-5, IL-9, IL-10 and IL-13), and growth factors (FGF-basic, PDGF, VEGF, G-CSF, IL-7 and IL-2) shortly after the first month compared to the pre-vaccination time point (Figure 6). Furthermore, most of the analyzed biomarkers showed a significant increase between 1 and 6 months, except for the pro-inflammatory cytokine IL-12, which exhibited a decrease between the third and sixth month of the study follow-up.

**Figure 7.**
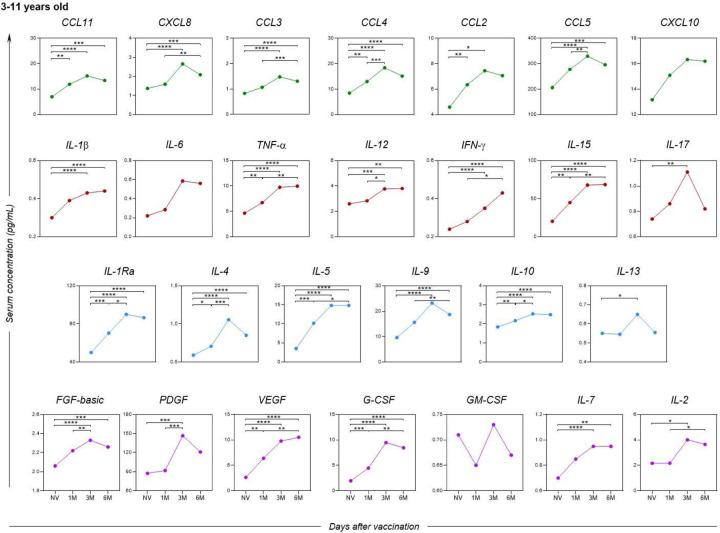
Kinetics of serum soluble mediators at one month (1M), three months (3M), and six months (6M) after receiving two doses of the CoronaVac vaccine in children (3–11 years old) as compared to the pre-vaccination period (Not Vaccinated - NV). The biomarkers were individually presented for chemokines (CXCL8, CCL11, CCL3, CCL4, CCL2, CCL5 and CXCL10); pro-inflammatory cytokines (IL-1β, IL-6, TNF-α, IL-12, IFN-γ, IL-15, IL-17); regulatory cytokines (IL-1Ra, IL-4, IL-5, IL-9, IL-10 and IL-13); and growth factors (FGF-basic, PDGF, VEGF, G-CSF, GM-CSF, IL-7 and IL-2). Statistical differences by Mann-Whitney and ANOVA with significance levels of p < 0.05 are denoted by (*) between groups.

When analyzing the soluble serum biomarkers separately by age group, a similar profile was observed among children (Figure 7) in comparison to the overall assessment of study participants, except for the biomarkers CXCL10 and IL-6, which did not show a statistically significant difference during the evaluated follow-up period.

**Figure 8.**
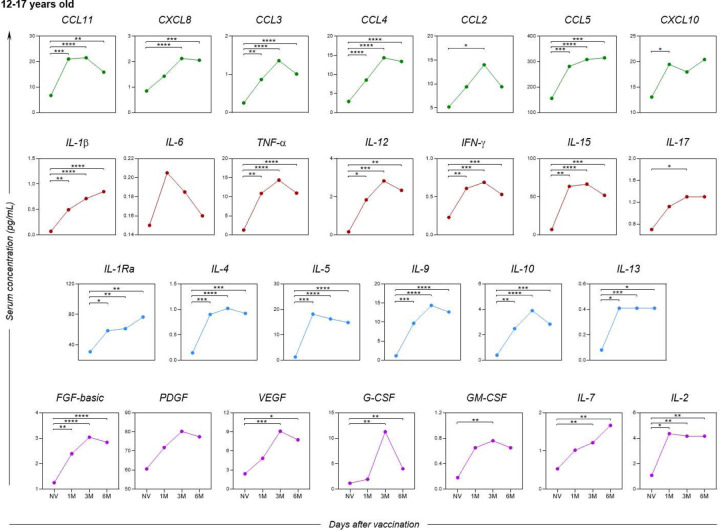
Kinetics of serum soluble mediators at one month (1M), three months (3M), and six months (6M) after receiving two doses of the CoronaVac vaccine in adolescents (12–17 years old) as compared to the pre-vaccination period (Not Vaccinated - NV). The biomarkers were individually presented for chemokines (CXCL8, CCL11, CCL3, CCL4, CCL2, CCL5 and CXCL10); pro-inflammatory cytokines (IL-1β, IL-6, TNF-α, IL-12, IFN-γ, IL-15, IL-17); regulatory cytokines (IL-1Ra, IL-4, IL-5, IL-9, IL-10 and IL-13); and growth factors (FGF-basic, PDGF, VEGF, G-CSF, GM-CSF, IL-7 and IL-2). Statistical differences by Mann-Whitney and ANOVA with significance levels of p < 0.05 are denoted by (*) between groups.

As for adolescents (Figure 8), apart from the inflammatory cytokine IL-6, there was no statistically significant difference in the growth factor PDGF between any of the post-vaccination time points.

**Figure 9.**
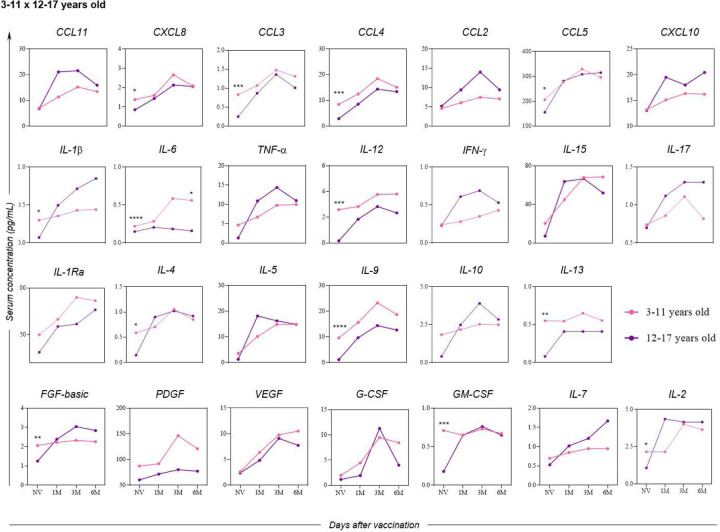
Differences in the kinetics of serum soluble mediators between children (3–11 years old) and adolescents (12–17 years old) at one month (1M), three months (3M), and six months (6M) after receiving two doses of the CoronaVac vaccine as compared to the pre-vaccination period (Not Vaccinated - NV). The biomarkers were individually presented for chemokines (CXCL8, CCL11, CCL3, CCL4, CCL2, CCL5 and CXCL10); pro-inflammatory cytokines (IL-1β, IL-6, TNF-α, IL-12, IFN-γ, IL-15, IL-17); regulatory cytokines (IL-1Ra, IL-4, IL-5, IL-9, IL-10 and IL-13); and growth factors (FGF-basic, PDGF, VEGF, G-CSF, GM-CSF, IL-7 and IL-2). Statistical differences by Mann-Whitney and ANOVA with significance levels of p < 0.05 are denoted by (*) between groups.

Comparing the cellular response between the age groups of 3–11 years and 12–17 years (Figure 9), significant higher levels of serum soluble mediators can be observed in children compared to adolescents, especially in the initial follow-up period of the study, except for the cytokine IL-6, which showed a statistically significant difference between these age groups after six months from the administration of the second dose of the CoronaVac vaccine.

Hence, it is evident that a robust and enduring cellular immune response, characterized by a combination of pro-inflammatory and regulatory elements, persists for up to six months after the administration of two doses of the CoronaVac vaccine in the children and adolescents observed within the scope of this study.

### 3.3 Safety Response - Identified AEs and SAEs

One hundred and ninety-two participants (192, 30%) presented AEs, resulting in a total of three hundred seventy-nine (379) events, with three of them occurring in infants (Table 2). Among these, 39.3% (149) were described as solicited systemic adverse events, 33% (125) were unsolicited adverse events, and 26.6% (101) were solicited local adverse events.

Among the major adverse events reported by study participants, 24% (91) had pain at the vaccine administration site, 8.7% (33) had fever, and 4.2% (16) had a runny nose, with the majority of these being of intensity grade 1 (69.9%, 265).

Regarding the causal relationship of adverse events with the vaccine, 29% (110) were not related, 26.4% (100) were certain, 24% (91) were unlikely, 13.2% (50) were possible, and 6.3% (24) were probable, with most individuals not requiring medical and/or hospital care (75.7%, 287).

The recorded adverse events were mild and transient, except for seven Serious Adverse Events (SAEs) (Table 3), which were monitored by the study’s clinical team until resolution. Among the SAEs, three were unlikely to be related to the vaccine, and four were classified as not related. All participants had a favorable recovery outcome.

### 3.4 Identification of SARS-CoV-2 by NGS

Active surveillance revealed two hundred and nine (209; 32.66%) suspected cases of SARS-CoV-2 infection, out of which fifty-six (56; 8.75%) were confirmed through RT-qPCR. There were no reports of hospitalizations or deaths. Eleven samples were submitted for sequencing (due to the Ct limit), all of which identified the Omicron variant, with 3 (27.27%) classified as Omicron (BA.2-like), 1 (9.09%) as Omicron (BA.5-like), and 7 (63.64%) as Omicron (Unassigned) (Tables 4 and 5).

## DISCUSSION

Data pertaining to immunogenicity, as assessed through total antibody levels by anti-SARS-CoV-2 ELISA, revealed the majority of children and adolescents exhibited seropositivity for anti-S IgG prior to vaccination, while 46% displayed seropositivity for anti-N IgG, indicating prior exposure to SARS-CoV-2 before immunization.

Epidemiological data published during the period of this study demonstrated peaks of COVID-19 transmission in Brazil, particularly in the southeastern region of the country where there was a prevalence of 58.4% of SARS-CoV-2 infections among severe acute respiratory syndrome cases, according to data published by InfoGripe. (INFOGRIPE, 2023). Furthermore, a study that assessed the circulation of SARS-CoV-2 in children in Brazil from April 2020 to July 2022 reported a higher risk of infection in children from symptomatic family adults, usually the mother, reinforcing the importance of vaccination across all age groups (FULLER et al., 2023).

Still, a comprehensive statistical analysis of the collective data indicated a significant rise in titers of anti-S IgG antibodies and anti-N IgG antibodies, as well as in seropositivity rates, one month after completing the primary immunization course with two doses of the CoronaVac vaccine. Similar findings were reported by Fernandes et al. (2021), wherein all children in the study exhibited a substantial increase in antibody titers induced by the CoronaVac vaccine one month after vaccination, underscoring a robust serological response to a single dose of CoronaVac in a pediatric population up to five years old, with no reports of severe adverse effects.

Despite a reduction in the overall mean antibody titers observed at three months post-vaccination in our study, these averages remained consistently high (above the detection limit of the reference assay) throughout the study period, demonstrating the durability and persistence of the total antibody response. The seropositivity rate reached 90% for anti-S IgG at the third month and 95% after six months of follow-up. Similarly, seropositivity rates reached 93% and 99% for anti-N IgG at the third- and sixth-months post-vaccination. Evaluation of the geometric mean titers of anti-S and anti-N IgG antibodies among participants grouped by age revealed a consistent pattern of seropositivity, confirming a substantial increase in total antibodies against SARS-CoV-2 within the first month after vaccination, followed by a stable immune response over the course of the six-month study.

These outcomes align with those reported by Rosa et al. (2022), indicating that 97% of adolescents between the ages of 11 and 17 displayed anti-S IgG antibodies against SARS-CoV-2 after receiving two doses of the CoronaVac vaccine. Moreover, the same adolescents were assessed for anti-N IgG as secondary immunogenicity markers, revealing a high seropositivity rate of 98%. Additionally, in accordance with the findings from the Immunita-02 study, previously published data indicated that over 96% of children and adolescents aged 3 to 17 years exhibited specific antibodies to SARS-CoV-2 after 28 days of receiving two doses of the CoronaVac vaccine (HAN et al., 2021).

Virus neutralization assays showed that a significant percentage of children and adolescents displayed seropositivity to B.1 lineage prior to vaccination. This percentage increased after the first dose, and it continued to rise following the second dose of the CoronaVac vaccine. When it comes to neutralizing antibodies to Omicron variant, most of the children and adolescents being monitored showed seropositivity before their first dose, indicating previous infection. After vaccination, they maintained significant seropositivity over time, indicating a sustained neutralizing response to Omicron variant across the different age groups. Good neutralizing antibody responses were also noted following the administration of the CoronaVac vaccine in previously published phase 1 and 2 studies, which reported the vaccine’s good tolerability and safety, along with satisfactory humoral responses in children and adolescents aged 3 to 17 years. Additionally, in our study, it was observed that the neutralizing antibody titers induced by the 3.0 μg dose exceeded those of the 1.5 μg dose. These findings support the use of the 3.0 μg dose of CoronaVac vaccine in a two-dose immunization regimen for further investigations in the same age group (HAN et al., 2021).

We chose to use individuals without a history of prior infection for the VNT assays. However, our data show that a significant portion of them already had neutralizing antibodies before vaccination, to both for Omicron and the ancestral B lineage, indicating previous, likely asymptomatic exposure. A meta-analysis study published in the Journal of Medical Virology compiled data on asymptomatic infections, revealing that during waves of Omicron variant infections, children and adolescents had a high proportion of asymptomatic cases (82%), while in the elderly (already widely vaccinated at that time), this proportion was lower (62%). These data reinforce the importance of vaccination among children and adolescents, as they may contribute to the ongoing circulation of SARS-CoV-2 (YU et al., 2022).

Regarding the cellular response data demonstrated in this study, in general, a similar profile was observed among the age groups, with occasional differences in pro-inflammatory cytokines when comparing the profiles of children to adolescents from the third to the sixth month of follow-up. An initial inflammation observed triggered by vaccination is a premise for the development of a robust cellular response. It is noticeable that there was an increasing release of soluble pro-inflammatory and regulatory mediators in the first three months post-vaccination, with the maintenance of the response at six months of post-vaccination follow-up.

Promising results regarding the cellular immune response in children and adolescents who received the CoronaVac vaccine have also been reported, as described by Soto and collaborators (2022). Their findings highlighted a significant increase in CD4^+^ AIM^+^ T-cells in response to the structural proteins of the virus. In the 12 to 17-year-old group, there was a notable expansion in the activation of CD4^+^ T-cells in response to all the structural proteins of SARS-CoV-2, indicating that the CoronaVac, as a inactivated virus vaccine, stimulates cellular immunity not only against the Spike (S) protein but also against the membrane (M) and nucleocapsid (N) proteins. Furthermore, an increase in IL-2 secretion in response to the S and N proteins was observed in both age groups, with this increase being particularly pronounced in individuals aged 12 to 17 years in relation to the M protein. The vaccine also promoted a pro-inflammatory profile, with an increase in IFN-γ and no increase in IL-4, in both the 3 to 11-year-old group and the 12 to 17-year-old group. Additionally, an increase in the frequency of memory CD4^+^ AIM^+^ T-cells in response to the SARS-CoV-2 proteins was noted, with a slight intensification observed in individuals aged 12 to 17 years, suggesting that the vaccine may induce long-lasting CD4^+^ T-cell responses (SOTO et al., 2022).

Among the 640 children and adolescents monitored, one third presented suspected cases of COVID-19. After conducting RT-qPCR tests, 56 cases of SARS-CoV-2 infections were confirmed, with no instances of moderate or severe COVID-19 necessitating hospitalization among the pediatric population. Of the swab samples collected from COVID-19 positive participants, 11 met the criteria for NGS sequencing, all of which were identified as belonging to the Omicron variant sub-lineages, including BA.2 and BA.5.

After vaccination, mostly mild and transient adverse effect were reported, being pain at the injection site the most common issue. The few serious adverse events observed were unrelated to the vaccine and included conditions like acute gastroenteritis, cellulitis, fractures, and asthmatic bronchiolitis.

Similar outcomes were observed in phase 1 and 2 studies described by Han and colleagues (2021), where most post-vaccination adverse reactions were mild and transient, with pain at the injection site being the most frequently reported event (73.13% of 550 participants). Furthermore, in a phase 3 study conducted in Chile, the primary adverse event reported after the first and second doses was also pain at the injection site. This study also concluded that CoronaVac was safe and immunogenic in individuals aged 3 to 17 years, inducing the production of neutralizing antibodies and activation of CD4^+^ T-cells to SARS-CoV-2 variants (SOTO et al., 2022).

Recently published data on the effectiveness of the CoronaVac vaccine against COVID-19 in Brazilian children (6 to 11 years), during a period of high Omicron variant circulation (January 21, 2022, to April 15, 2022), indicated an estimated vaccine effectiveness of 39.8% (95% CI 33.7–45.4) against symptomatic infection at ≥14 days post-second dose. For hospitalization, vaccine effectiveness was 59.2% (95% CI 11.3–84.5) at ≥14 days (FLORENTINO et al., 2022). Conversely, noteworthy results emerged from the evaluation of CoronaVac vaccine effectiveness in Chile, involving a large national prospective cohort of approximately two million children and adolescents aged 6 to 16 years. The estimated effectiveness of the CoronaVac vaccine stood at 74.5% (95% CI, 73.8–75.2), 91.0% (95% CI, 87.8–93.4), and 93.8% (95% CI, 87.8–93.4) for the prevention of COVID-19, hospitalization, and ICU admission, respectively. For children aged 6 to 11 years, vaccine effectiveness was 75.8% (95% CI, 74.7–76.8) against COVID-19 and 77.9% (95% CI, 61.5–87.3) against hospitalization (JARA et al., 2023).

Another study assessing CoronaVac vaccine effectiveness in children aged 3 to 5 years, conducted during the Omicron outbreak in Chile, revealed estimated effectiveness of 38.2% (95% CI, 36.5–39.9) against symptomatic COVID-19, 64.6% (95% CI, 49.6–75.2) against hospitalization, and 69.0% (95% CI, 18.6–88.2) against ICU admission. The study concluded that despite the modest effectiveness against symptomatic COVID-19, CoronaVac vaccination provided robust protection against severe COVID-19 in this age group (JARA et al., 2022).

## CONCLUSION

In conclusion, the data evaluation from this study underlines a robust immunogenic response elicited by the CoronaVac vaccine (Sinovac/Butantan Institute) and its sustained nature in children and adolescents aged 3 to 17 years over a period of up to six months following the completion of the primary vaccination regimen. The vaccine notably and comprehensively stimulated the production of specific IgG antibodies targeting the S and N proteins of the virus, as well as the generation of neutralizing antibodies effective to both the B.1 lineage and the Omicron variant (BA.1). Furthermore, this vaccination regimen triggered a substantial mixed-pattern cellular response with widespread release of pro-inflammatory and regulatory mediators, highlighting the vaccine’s safety and efficacy in mitigating COVID-19 in children and adolescents.

## Figures and Tables

**Table 1.** Demographic data of participants in the study.

Age, years	N	%
3–11	562	87.81
12–17	78	12.19
Gender	N	%
Male	325	50.78
Female	315	49.22
Locality, home city	N	%
Serrana, São Paulo, Brazil	547	85.47
Belo Horizonte, Minas Gerais, Brazil	93	14.53
COVID-19 prior to vaccination	N	%
Yes	88	13.75
No	552	86.25
Main reported comorbidities	N	%
Rhinitis	73	11.41
Asthma	42	6.56
Obesity	18	2.81
Sinusitis	9	1.41
Bronchiolitis	8	1.25
Bronchitis	8	1.25

**Table 2.** Adverse events reported after the administration of the CoronaVac vaccine in children and adolescents during a six-month follow-up.

Adverse event reports	N	%
Total number of participants with reports	192	30.0
Total adverse events reported	379	
Type of adverse event	N	%
Solicited systemic adverse event	149	39.3
Unsolicited adverse event	125	33.0
Requested local adverse event	101	26.6
Not classified	4	1.1
Main adverse events reported	N	%
Pain at the vaccine administration site	91	24.0
Covid-19	47	12.4
Fever	33	8.7
Cough	24	6.3
Runny nose	16	4.2
Cold	16	4.2
Symptoms of flu syndrome	15	4.0
Vomiting	10	2.6
Sore throat	6	1.6
Diarrhea	6	1.6
Sinusitis	5	1.3
Acute tonsillitis	4	1.1
Otitis	4	1.1
Odynophagia	3	0.8
Abdominal pain	2	0.5
Edema at the vaccine administration site	2	0.5
Malaise	2	0.5
Nausea	2	0.5
Allergy	2	0.5
Classification of the intensity of the adverse event	N	%
1	265	69.9
2	88	23.2
3	11	2.9
4	8	2.1
Not classified	7	1.8
Causal relationship of the adverse event with the vaccine	N	%
Not related	110	29.0
Certain	100	26.4
Unlikely	91	24.0
Possible	50	13.2
Probable	24	6.3
Not classified	4	1.1
Need for medical attention	N	%
No	287	75.7
Yes	89	23.5
Not described	3	0.8

**Table 3.** Serious adverse events reported after the administration of the CoronaVac vaccine in children and adolescents during a six-month follow-up.

Local	Serious adverse event	Causality	Predictability	Severity Criterion	Clinical outcome
Serrana, SP	Acute Gastroenteritis	Unlikely	Not expected	Hospitalization	Recovered
Serrana, SP	Pre and post septal cellulite on the right	Unlikely	Not expected	Hospitalization	Recovered
Serrana, SP	Arm fracture	Not related	Not expected	Hospitalization	Recovered
Serrana, SP	Cellulitis	Not related	Not expected	Hospitalization	Recovered
Serrana, SP	Arm fracture	Not related	Not expected	Hospitalization	Not recovered
Belo Horizonte, MG	Asthmatic bronchiolitis	Unlikely	Not expected	Hospitalization	Recovered
Belo Horizonte, MG	Mastoiditis	Not related	Not expected	Hospitalization	Recovered

**Table 4.** Data on infections, hospitalizations, and deaths among study participants.

Infections, hospitalizations, and deaths	N	%
Monitoring children and adolescents	640	100
Suspected infection	209	32.66
Confirmed COVID-19 diagnosis	56	8.75
Hospitalizations	0	0.00
Deaths	0	0.00

**Table 5.** Identification of SARS-CoV-2 variant obtained by NGS among study participants.

Identification of SARS-CoV-2 by NGS	N	%
Next generation sequencing (NGS) performed	11	100
Omicron (BA.2-like)	3	27,27
Omicron (BA.5-like)	1	9,09
Omicron (Unassigned)	7	63,64

## Data Availability

The datasets used and/or analysed during the current study available from the corresponding author on reasonable request.
